# Supplementary material for: A geospatial analysis of accessibility and availability to implement the primary healthcare roadmap in Ethiopia
Source: Commun Med (Lond). 2023 Oct 7;3:140. doi: 10.1038/s43856-023-00372-z (PMC10560263; doi:10.1038/s43856-023-00372-z)
Supplement: Supplementary file 1 — Supplementary material [file 43856_2023_372_MOESM1_ESM.pdf]

A geospatial analysis of accessibility and availability to implement the primary healthcare roadmap in Ethiopia

Fleur Hierink[1,2], Olusola Oladeji[3], Ann Robins[4], Maria F Muñiz[5], Yejimmawerk Ayalew[6], Nicolas Ray[1,2]

1. GeoHealth group, Institute of Global Health, University of Geneva, Geneva, Switzerland
2. Institute for Environmental Sciences, University of Geneva, Geneva, Switzerland
3. UNICEF Belize, Belize City, Belize
4. UNICEF Uganda, Country Office, Kampala, Uganda
5. UNICEF, Eastern and Southern Africa Regional Office, Nairobi, Kenya
6. UNICEF Nigeria Country Office, Abuja, Nigeria

Supplementary information

Supplementary Table 1 – Motorized & walking travel scenario for the accessibility analysis. Motorized transport is considered on roads and walking speeds are considered off-road.

Label	Speed	Mode
Tree cover	2	Walking
Shrub cover	3	Walking
Grassland	4	Walking
Cropland	3	Walking
Vegetation Aquatic	2	Walking
Lichen Mosses	2	Walking
Bare Area	1.5	Walking
Built-up area	2.5	Walking
Open Water	1	Walking
Asphalt	60	Motorized
Gravel	40	Motorized
Earth	30	Motorized

Supplementary Table 2 – Walking travel scenario for the accessibility analysis. Walking speeds are considered on all different types of surfaces, including roads.

Label	Speed	Mode
Tree cover	2	Walking
Shrub cover	3	Walking
Grassland	4	Walking
Cropland	3	Walking
Vegetation Aquatic	2	Walking
Lichen Mosses	2	Walking
Bare Area	1.5	Walking
Built-up area	2.5	Walking
Open Water	1	Walking
Asphalt	4	Walking
Gravel	4	Walking
Earth	4	Walking

Supplementary Table 3 – Health workforce requirements per health center and health worker group as set by the Ethiopian government.

Health Worker Group	Required number of employees
Drugist	1
General Practitioner	1
Health Information Technician	1
Health Officer	2
Laboratory Degree	1
Laboratory Diploma	1
Midwife Degree	2
Midwife Diploma	3
Nurse	5
Pharmacist	1
Public Health Nurse	1

Supplementary Table 4 – Health workforce requirements as set out by the World Health Organization[17] to reach at least 70% universal health coverage and the found densities in Somali region, Ethiopia. Densities reflect the number of health workers per 10.000 people living within 1 or 2 hours of a health center.

	Density 10 000 population for at least 70% UHC service coverage index as adapted from the WHO	Density 10 000 population in 1-hour catchment of health centers as found in this study	Density 10 000 population in 2-hour catchment of health centers as found in this study
Medical doctors (generalist and specialist medical practitioners)	7.77	0.13	0.08
Nursing and midwife professionals (nursing and midwifery associate professionals)	58.64	13.86	8.64
Pharmacists, pharmaceutical technicians and assistants	14.72	13.86	8.64
Medical assistants	0.90	1.84	1.15
Medical and pathology laboratory technicians	14.00	1.53	0.95
Community Health Workers	25.20	-	-

Supplementary Table 5 – Reclassification of the Ethiopian health worker groups according to the WHO workforce classes.

Ethiopian health worker classification	Reclassification according to WHO workforce classes
General practitioner	Medical doctor
Health officer	Medical assistants
Nurse bachelor	Nursing and midwife professionals
Nurse diploma	Nursing and midwife professionals
Midwife diploma	Nursing and midwife professionals
Midwife degree	Nursing and midwife professionals
Laboratory diploma	Laboratory technicians
Laboratory degree	Laboratory technicians
Drugist	Pharmacists
Pharmacy	Pharmacists
Public health nurse	Nursing and midwife professionals
Health information technician	-

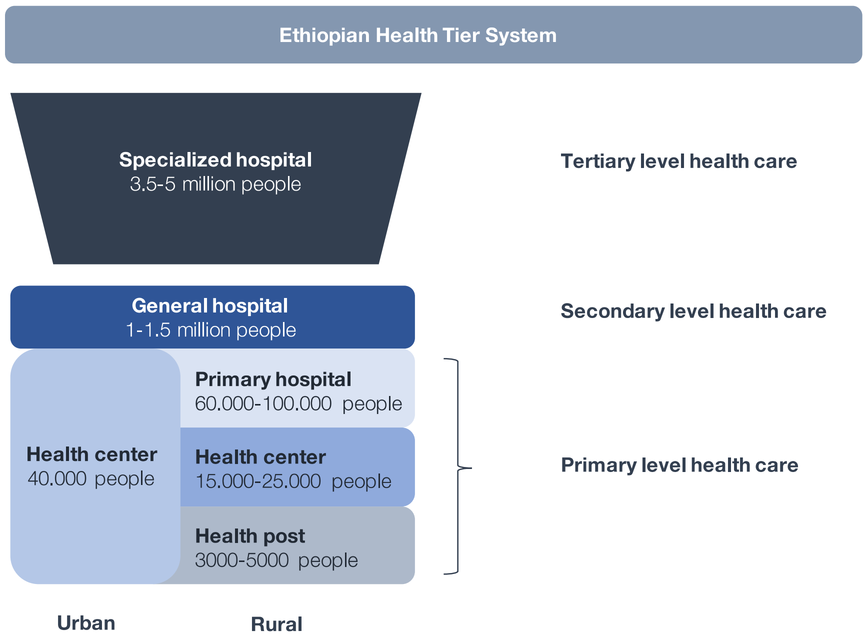

Supplementary Figure 1 – Overview of the Ethiopian health tier system and the national coverage benchmarks per facility type. Numbers indicate the approximate population they cover. Adapted from the Ethiopian Health Sector Development Plan (2016-2020).

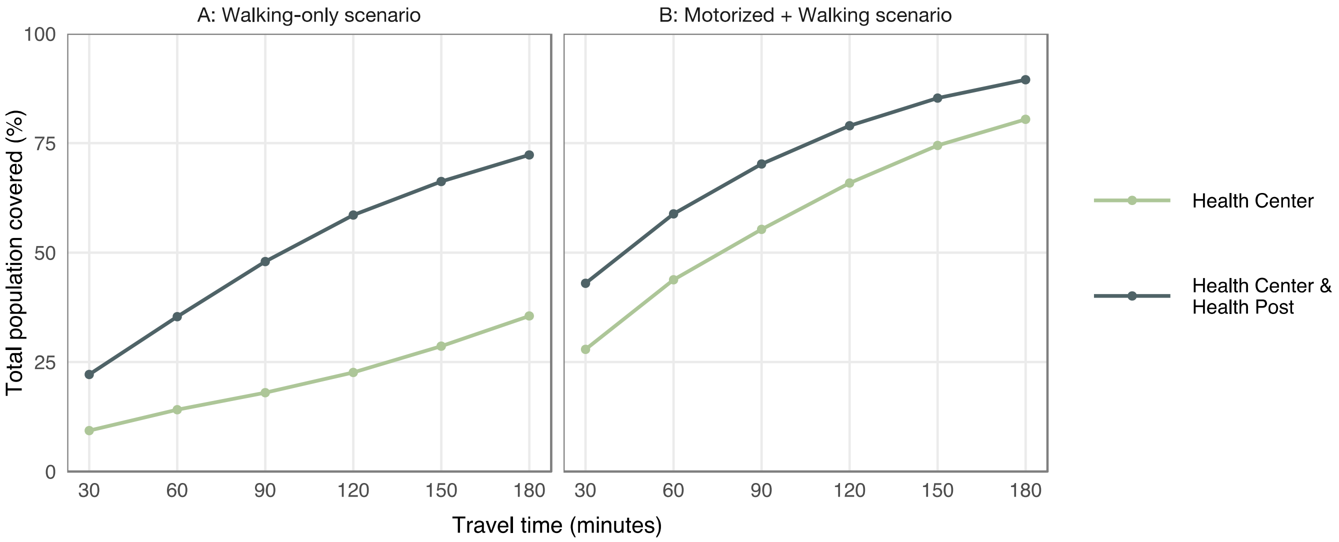

Supplementary Figure 2 – The proportion of the population covered by health centers and health posts in 30, 60, 90, 120, 150, and 180 minutes travel time considering (A) a walking-only scenario and (B) a motorized and walking scenario using motorized speeds on road and walking speeds off road.

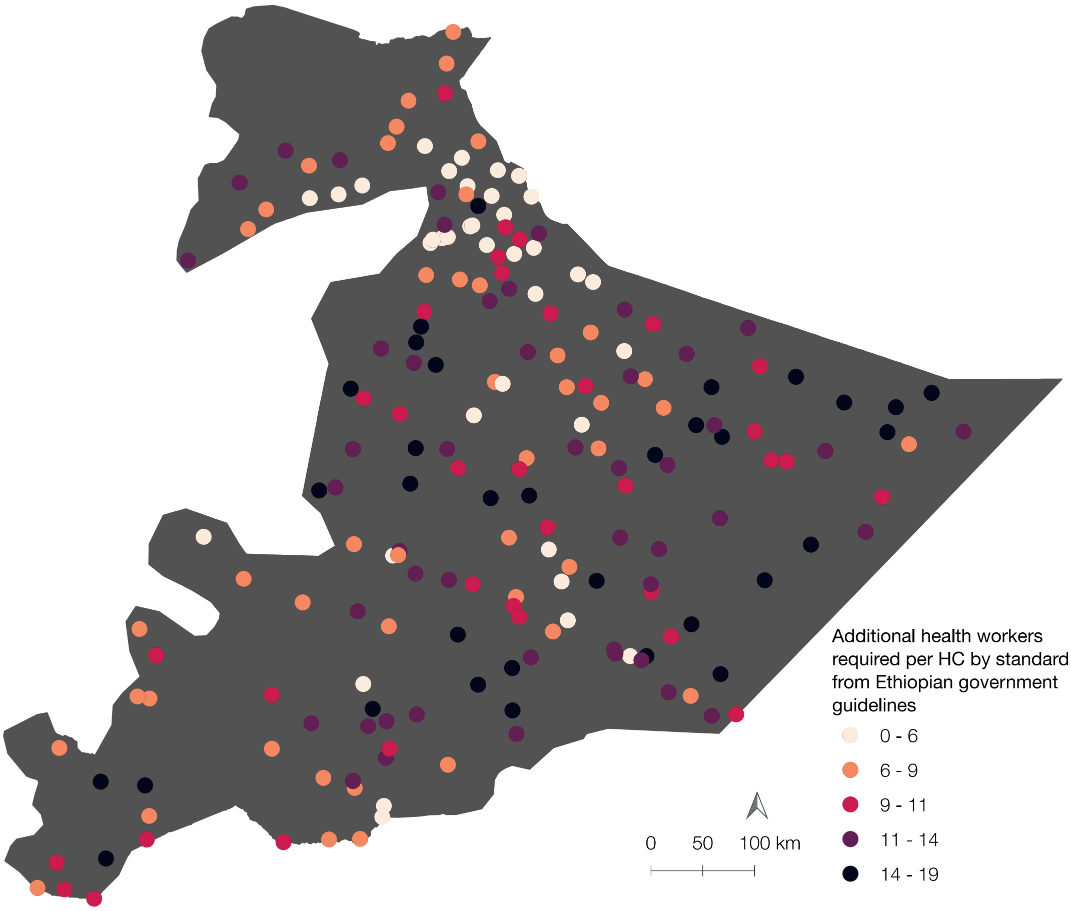

Supplementary Figure 3 – Geographical distribution showing the location of health centers in Somali region and the additional health workers required per health center as outlined in the Ethiopian government guidelines.

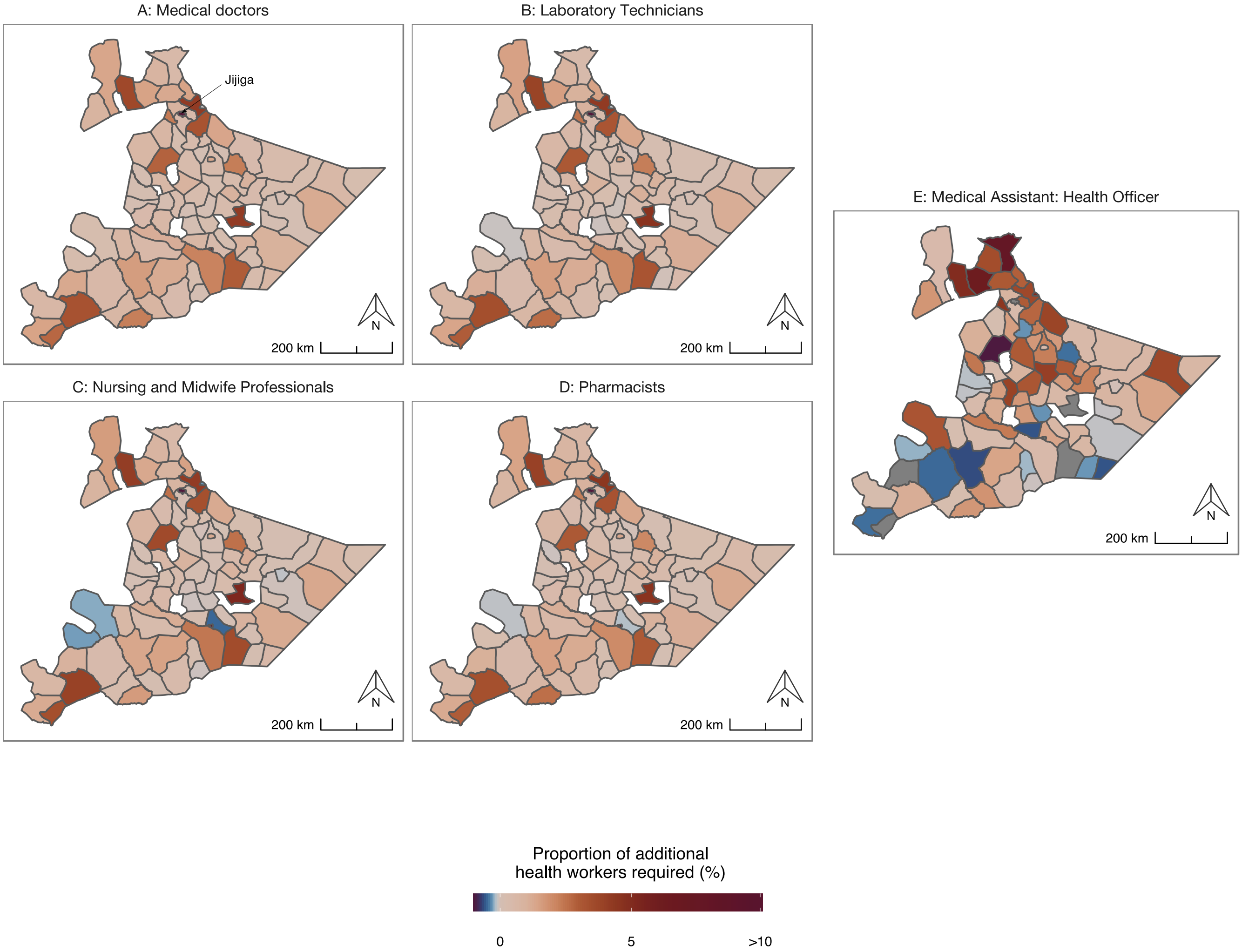

Supplementary Figure 4 – Maps showing where the required number of health workers to reach the WHO benchmark[18] is proportionally highest in 1-hour catchments. Each map represents a specific health worker group; (a) medical doctors, (b) laboratory technicians, (c) nursing and midwife professionals, (d) pharmacists, (e) medical assistants. Red colors indicate a proportionally higher need of additional health workers. Blue indicates that the density of health workers is higher than required under the WHO benchmark.

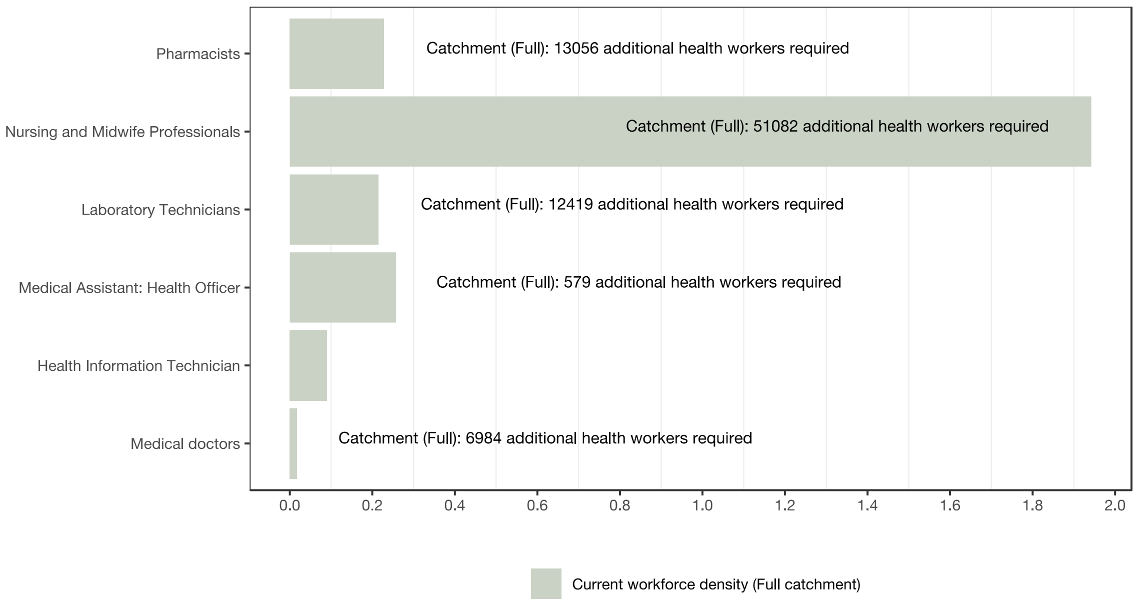

Supplementary Figure 5 – Health worker densities in health centers in full catchments compared against the WHO required workforce density benchmarks[18]. Filled bars indicate the found densities full catchments. Text represents the additional number of health workers needed per health worker group to achieve the WHO required workforce density.
