# Supplementary material for: A geospatial analysis of accessibility and availability to implement the primary healthcare roadmap in Ethiopia
Source: Commun Med (Lond). 2023 Oct 7;3:140. doi: 10.1038/s43856-023-00372-z (PMC10560263; doi:10.1038/s43856-023-00372-z)
Supplement: Supplementary file 7 — Description of Additional Supplementary Files [file 43856_2023_372_MOESM7_ESM.pdf]

Description of Additional Supplementary Files

File Name: Supplementary Data 1

Description: Source data for Figure 2

File Name: Supplementary Data 2

Description: Source data for Figure 3

File Name: Supplementary Data 3

Description: Source data for Figure 4a

File Name: Supplementary Data 4

Description: Source data for Figure 4b

File Name: Supplementary Data 5

Description: Source data for Figure 5
