# Supplementary material for: A geospatial analysis of accessibility and availability to implement the primary healthcare roadmap in Ethiopia
Source: Commun Med (Lond). 2023 Oct 7;3:140. doi: 10.1038/s43856-023-00372-z (PMC10560263; doi:10.1038/s43856-023-00372-z)
Supplement: Supplementary file 9 — Reporting Summary [file 43856_2023_372_MOESM9_ESM.pdf]

nature portfolio

Corresponding author(s): Fleur Hierink

Last updated by author(s): Sep 20, 2023

Reporting Summary

Nature Portfolio wishes to improve the reproducibility of the work that we publish. This form provides structure for consistency and transparency in reporting. For further information on Nature Portfolio policies, see our Editorial Policies and the Editorial Policy Checklist.

Statistics

For all statistical analyses, confirm that the following items are present in the figure legend, table legend, main text, or Methods section.

n/a	Confirmed	
☒	☐	The exact sample size (*n*) for each experimental group/condition, given as a discrete number and unit of measurement
☒	☐	A statement on whether measurements were taken from distinct samples or whether the same sample was measured repeatedly
☒	☐	The statistical test(s) used AND whether they are one- or two-sided<br>*Only common tests should be described solely by name; describe more complex techniques in the Methods section.*
☒	☐	A description of all covariates tested
☒	☐	A description of any assumptions or corrections, such as tests of normality and adjustment for multiple comparisons
☒	☐	A full description of the statistical parameters including central tendency (e.g. means) or other basic estimates (e.g. regression coefficient) AND variation (e.g. standard deviation) or associated estimates of uncertainty (e.g. confidence intervals)
☒	☐	For null hypothesis testing, the test statistic (e.g. *F*, *t*, *r*) with confidence intervals, effect sizes, degrees of freedom and *P* value noted<br>*Give P values as exact values whenever suitable.*
☒	☐	For Bayesian analysis, information on the choice of priors and Markov chain Monte Carlo settings
☒	☐	For hierarchical and complex designs, identification of the appropriate level for tests and full reporting of outcomes
☒	☐	Estimates of effect sizes (e.g. Cohen's *d*, Pearson's *r*), indicating how they were calculated

Our web collection on statistics for biologists contains articles on many of the points above.

Software and code

Policy information about availability of computer code

Data collection	The data used in this study include digital elevation model (DEM) from the Shuttle Radar Topography Mission (SRTM), landcover data from Sentinel-1, road data from OpenStreetMap and the Ethiopian Federal Road Authority, hydrography data from OpenStreetMap, population distribution data from WorldPop, as well as health facility and human resource data obtained from UNICEF and the Regional Health Bureau in Somali Region, Ethiopia. The datasets related to health facility location and staffing sufficiency are not publicly available due to the sensitivity and nature of the information but are available from the corresponding author on reasonable request. All other data sources are openly available and can be downloaded from the respective sources. Source data for the figures are available as Supplementary Data 1-5 and can also be downloaded from Zenodo (https://doi.org/10.5281/zenodo.8362737).
Data analysis	The analysis was done using the software AccessMod version 5. The results were then analysed and visualized using R version 4.1.1 and QGIS version 3.22.

For manuscripts utilizing custom algorithms or software that are central to the research but not yet described in published literature, software must be made available to editors and reviewers. We strongly encourage code deposition in a community repository (e.g. GitHub). See the Nature Portfolio guidelines for submitting code & software for further information.

Data

Policy information about availability of data

All manuscripts must include a data availability statement. This statement should provide the following information, where applicable:

- Accession codes, unique identifiers, or web links for publicly available datasets
- A description of any restrictions on data availability
- For clinical datasets or third party data, please ensure that the statement adheres to our policy

The data used in this study include digital elevation model (DEM) from the Shuttle Radar Topography Mission (SRTM), landcover data from Sentinel-1, road data from OpenStreetMap and the Ethiopian Federal Road Authority, hydrography data from OpenStreetMap, population distribution data from WorldPop, as well as health facility and human resource data obtained from UNICEF and the Regional Health Bureau in Somali Region, Ethiopia. The datasets related to health facility location and staffing sufficiency are not publicly available due to the sensitivity and nature of the information but are available from the corresponding author on reasonable request. All other data sources are openly available and can be downloaded from the respective sources. Source data for the figures are available as Supplementary Data 1-5 and can also be downloaded from Zenodo (https://doi.org/10.5281/zenodo.8362737).

Human research participants

Policy information about studies involving human research participants and Sex and Gender in Research.

Reporting on sex and gender	*Use the terms sex (biological attribute) and gender (shaped by social and cultural circumstances) carefully in order to avoid confusing both terms. Indicate if findings apply to only one sex or gender; describe whether sex and gender were considered in study design whether sex and/or gender was determined based on self-reporting or assigned and methods used. Provide in the source data disaggregated sex and gender data where this information has been collected, and consent has been obtained for sharing of individual-level data; provide overall numbers in this Reporting Summary. Please state if this information has not been collected. Report sex- and gender-based analyses where performed, justify reasons for lack of sex- and gender-based analysis.*
Population characteristics	*Describe the covariate-relevant population characteristics of the human research participants (e.g. age, genotypic information, past and current diagnosis and treatment categories). If you filled out the behavioural & social sciences study design questions and have nothing to add here, write "See above."*
Recruitment	*Describe how participants were recruited. Outline any potential self-selection bias or other biases that may be present and how these are likely to impact results.*
Ethics oversight	*Identify the organization(s) that approved the study protocol.*

Note that full information on the approval of the study protocol must also be provided in the manuscript.

Field-specific reporting

Please select the one below that is the best fit for your research. If you are not sure, read the appropriate sections before making your selection.

☐ Life sciences ☐ Behavioural & social sciences ☒ Ecological, evolutionary & environmental sciences

For a reference copy of the document with all sections, see nature.com/documents/nr-reporting-summary-flat.pdf

Ecological, evolutionary & environmental sciences study design

All studies must disclose on these points even when the disclosure is negative.

Study description	Geospatial analysis coupling an assessment of geographical access to primary health care services and the availability of health professionals in the Somali region of Ethiopia.
Research sample	We had the geographic coordinates of health centers (n = 201) and health posts (n = 1049). For health posts we had information on the number of health extension workers available at each health posts. For health centers we had additional information on the availability of different health professionals. We calculated the geographic coverage of this primary health care network at the woreda level (n = 99).
Sampling strategy	N/A
Data collection	The data used in this study include digital elevation model (DEM) from the Shuttle Radar Topography Mission (SRTM), landcover data from Sentinel-1, road data from OpenStreetMap and the Ethiopian Federal Road Authority, hydrography data from OpenStreetMap, population distribution data from WorldPop, as well as health facility and human resource data obtained from UNICEF and the Regional Health Bureau in Somali Region, Ethiopia. The datasets related to health facility location and staffing sufficiency are not publicly available due to the sensitivity and nature of the information but are available from the corresponding author on reasonable

	request. All other data sources are openly available and can be downloaded from the respective sources. Source data for the figures are available as Supplementary Data 1-5 and can also be downloaded from Zenodo (https://doi.org/10.5281/zenodo.8362737).
Timing and spatial scale	Data analyses were run between in 2021 and 2022, however reference dates of the can date back further in time. The health facility data were originally collected in 2020 and covered Somali region in Ethiopia. The spatial data used as input layers to the accessibility analysis covered Ethiopia entirely, but were clipped to cover the Somali region in Ethiopia.
Data exclusions	No data was excluded from the analysis
Reproducibility	The openly available data that were used to calculate accessibility to primary health care services are available via the relevant sources as indicated above. The source data behind the figures and the code used to generate the figures are openly available through Zenodo (https://doi.org/10.5281/zenodo.8362737). AccessMod is open source software that can be used freely to reproduce the findings of this study.
Randomization	N/A
Blinding	N/A

Did the study involve field work? ☐ Yes ☒ No

Reporting for specific materials, systems and methods

We require information from authors about some types of materials, experimental systems and methods used in many studies. Here, indicate whether each material, system or method listed is relevant to your study. If you are not sure if a list item applies to your research, read the appropriate section before selecting a response.

Materials & experimental systems

n/a	Involved in the study
☒	☐ Antibodies
☒	☐ Eukaryotic cell lines
☒	☐ Palaeontology and archaeology
☒	☐ Animals and other organisms
☒	☐ Clinical data
☒	☐ Dual use research of concern

Methods

n/a	Involved in the study
☒	☐ ChIP-seq
☒	☐ Flow cytometry
☒	☐ MRI-based neuroimaging
